# Methyl 1-ethyl-7-methyl-4-oxo-1,4-di­hydro-1,8-naphthyridine-3-carboxyl­ate monohydrate

**DOI:** 10.1107/S1600536812004333

**Published:** 2012-02-10

**Authors:** Rehana Yasmeen, Muhammad Zia-ur-Rehman, Muhammad Azim Khattak, Muhammad Nadeem Arshad, Islam Ullah Khan

**Affiliations:** a Department of Chemistry, Gomal University, Dera Ismail Khan, Pakistan; b Applied Chemistry Research Centre, Pakistan Council of Scientific and Industrial Research Laboratories Complex, Lahore 54600, Pakistan; c Department of Chemistry, University of Gujrat 50781, Gujrat, Pakistan; d Materials Chemistry Laboratory, Department of Chemistry, GC University, Lahore 54000, Pakistan

## Abstract

In the structure of the title compound, C_13_H_14_N_2_O_3_·H_2_O, all atoms of the organic molecule except the terminal methyl group of the ethyl group attached to the N atom of the pyridinone ring are roughly coplanar, with an r.m.s. deviation of 0.0897 Å. In the crystal, C—H⋯O contacts link pairs of naphthyridine mol­ecules into head-to-tail dimers. These are joined by strong O—H⋯O hydrogen bonds from the water molecules into infinite chains along the *a* axis.

## Related literature

For the coordination properties of 1,8-naphthyridine ligands, see: Gavrilova & Bosnich (2004 ▶); Mintert & Sheldrick (1995 ▶). For their biological activity, see: Chen *et al.* (2001 ▶); Ferrarini *et al.* (2000 ▶); Roma *et al.* (2000 ▶). For related structures, see: Deeba, Khan, Zia-ur-Rehman, Çaylak & Şahin (2009 ▶); Deeba, Khan, Zia-ur-Rehman, Şahin & Çaylak (2009 ▶). For graph-set notation, see: Bernstein *et al.* (1995 ▶).
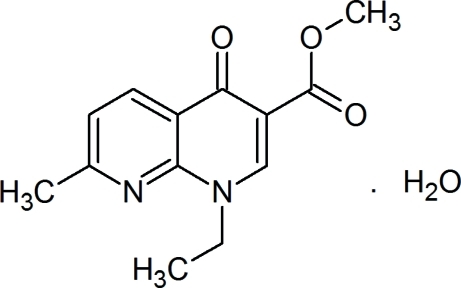


## Experimental

### 

#### Crystal data

C_13_H_14_N_2_O_3_·H_2_O
*M* *_r_* = 264.28
Monoclinic, 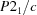

*a* = 4.6989 (1) Å
*b* = 23.7246 (7) Å
*c* = 11.3635 (3) Å
β = 91.646 (1)°
*V* = 1266.27 (6) Å^3^
*Z* = 4
Mo *K*α radiation
μ = 0.10 mm^−1^
*T* = 296 K
0.19 × 0.09 × 0.07 mm

#### Data collection

Bruker Kappa APEXII CCD diffractometer
12165 measured reflections
3128 independent reflections
2152 reflections with *I* > 2σ(*I*)
*R*_int_ = 0.026

#### Refinement

*R*[*F* ^2^ > 2σ(*F* ^2^)] = 0.047
*wR*(*F* ^2^) = 0.163
*S* = 0.98
3128 reflections
181 parameters
H atoms treated by a mixture of independent and constrained refinement
Δρ_max_ = 0.27 e Å^−3^
Δρ_min_ = −0.18 e Å^−3^

### 

Data collection: *APEX2* (Bruker, 2007 ▶); cell refinement: *SAINT* (Bruker, 2007 ▶); data reduction: *SAINT*; program(s) used to solve structure: *SHELXS97* (Sheldrick, 2008 ▶); program(s) used to refine structure: *SHELXL97* (Sheldrick, 2008 ▶); molecular graphics: *ORTEP-3 for Windows* (Farrugia, 1997 ▶) and *PLATON* (Spek, 2009 ▶); software used to prepare material for publication: *WinGX* (Farrugia, 1999 ▶) and *PLATON*.

## Supplementary Material

Crystal structure: contains datablock(s) I, global. DOI: 10.1107/S1600536812004333/sj5191sup1.cif

Structure factors: contains datablock(s) I. DOI: 10.1107/S1600536812004333/sj5191Isup2.hkl

Supplementary material file. DOI: 10.1107/S1600536812004333/sj5191Isup3.cml

Additional supplementary materials:  crystallographic information; 3D view; checkCIF report

## Figures and Tables

**Table 1:** Hydrogen-bond geometry (Å, °)

*D*—H⋯*A*	*D*—H	H⋯*A*	*D*⋯*A*	*D*—H⋯*A*
O4—H4*B*⋯O1^i^	1.01 (4)	2.02 (4)	2.994 (2)	163 (3)
O4—H4*A*⋯O1^ii^	0.84 (3)	2.09 (3)	2.928 (2)	176 (3)
O4—H4*B*⋯O3^i^	1.01 (4)	2.56 (3)	3.224 (2)	124 (2)
C3—H3⋯O2^iii^	0.93	2.40	3.293 (2)	160
C11—H11*C*⋯O4^iv^	0.96	2.59	3.539 (3)	168

Symmetry codes: (i) 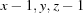; (ii) 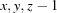; (iii) 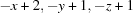; (iv) 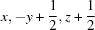.

**Fig. 1.**
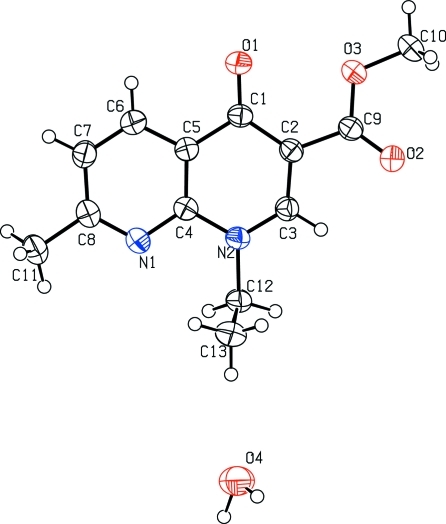
The structure of (I) with 50% displacement ellipsoids.

**Fig. 2.**
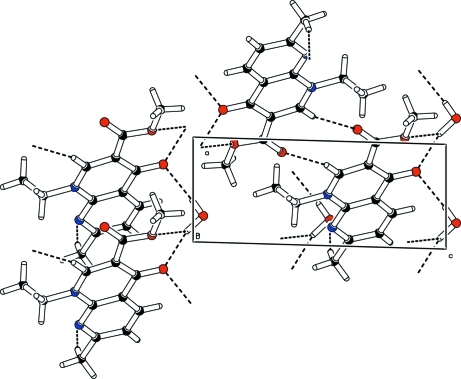
A crystal packing plot parallel to a with hydrogen bonds drawn as dashed lines.

## supplementary crystallographic information

### Comment

1,8-Naphthyridines have been cited in the literature for their interesting complexation properties due to the possibility of their bonding with metals in several coordination modes. These include monodendate, chelating bidendate and dinuclear bridging coordination (Gavrilova & Bosnich, 2004; Mintert & Sheldrick, 1995). These compounds are also biologically active with anti-bacterial (Chen *et al.*, 2001), anti-hypertensive (Ferrarini *et al.*, 2000) and anti-inflammatory (Roma *et al.*, 2000) properties. In a continuation of our work on the synthesis, biological activity and crystal structures of various 1,8-naphthyridines (Deeba, Khan, Zia-ur-Rehman, Çaylak & Şahin, 2009; Deeba, Khan, Zia-ur-Rehman, Şahin & Çaylak, 2009), we herein report the synthesis and crystal structure of the title compound (I) (Fig. 1; Scheme 1).

The two fused aromatic rings (C1/C2/C3/N2/C4/C5) & (C4/C5/C6/C7/C8/N1) are co-planar with root mean square (r. m. s.) deviations of 0.0103 Å & 0.0023 Å and are twisted at a dihedral angle of 1.20 (10)°. The methyl ester unit attached to the pyridinone ring is also planar with an r. m. s. deviation of 0.0051 Å and oriented at dihedral angles of 10.97 (15)° & 11.41 (15)° with respect to the pyridinone and pyridine rings respectively. In addition, a solvent water molecule is also present and stabilizes the crystal structure through intermolecular hydrogen bonding interactions. The molecule exhibits C—H···O type weak intermolecular hydrogen bonding and forms dimers through the formation of ten membered ring motif *R*_2_^2^(10) (Bernstein, *et al.*, 1995). These are further connected *via* water molecules along the *a* axis to form infinite chains. On the other hand, one of the hydrogen atoms of water molecule is also involved in the formation of six membered ring motif with O atoms of pyridinone ring and the ester (Fig. 2, Table. 1).

### Experimental

A mixture of 1-ethyl-7-methyl-4-oxo-1,4-dihydro-1,8-naphthyridine-3-carboxylic acid (100.0 mmol; 23.22 g) and thionyl chloride (50 ml) was refluxed for a period of 4 h followed by distillation (under reduced pressure) of the excess thionyl chloride. After complete removal of thionyl chloride, methanol (100 ml) was slowly added and stirred for two hours followed by the addition of ice cooled water (300 ml). The contents were washed with aqueous sodium carbonate (0.5 M) and water respectively followed by crystallization from methanol to give suitable crystals. Yield: 92%.

### Refinement

All C-bonded H-atoms were positioned in an idealized geometry, with C—H = 0.95Å for aromatic CH and C—H =0.98Å for the methyl group. U(H) was set to 1.2*U*_eq_ for all C_aromatic_ and 1.5*U*_eq_ for the C_methyl_ & oxygen atoms. The H atoms of the water molecule were located in a difference Fourier map and refined freely with U(H) = 1.5U_eq_(O).

### Crystal data

**Table:**

C_13_H_14_N_2_O_3_·H_2_O	*F*(000) = 560
*M**_r_* = 264.28	*D*_x_ = 1.386 Mg m^−^^3^
Monoclinic, *P*2_1_/*c*	Mo *K*α radiation, λ = 0.71073 Å
Hall symbol: -P 2ybc	Cell parameters from 3082 reflections
*a* = 4.6989 (1) Å	θ = 2.5–27.9°
*b* = 23.7246 (7) Å	µ = 0.10 mm^−^^1^
*c* = 11.3635 (3) Å	*T* = 296 K
β = 91.646 (1)°	Needle, white
*V* = 1266.27 (6) Å^3^	0.19 × 0.09 × 0.07 mm
*Z* = 4	

### Data collection

**Table:**

Bruker Kappa APEXII CCD diffractometer	2152 reflections with *I* > 2σ(*I*)
Radiation source: fine-focus sealed tube	*R*_int_ = 0.026
Graphite monochromator	θ_max_ = 28.3°, θ_min_ = 1.7°
φ and ω scans	*h* = −6→6
12165 measured reflections	*k* = −29→31
3128 independent reflections	*l* = −14→15

### Refinement

**Table:**

Refinement on *F*^2^	Primary atom site location: structure-invariant direct methods
Least-squares matrix: full	Secondary atom site location: difference Fourier map
*R*[*F*^2^ > 2σ(*F*^2^)] = 0.047	Hydrogen site location: inferred from neighbouring sites
*wR*(*F*^2^) = 0.163	H atoms treated by a mixture of independent and constrained refinement
*S* = 0.98	*w* = 1/[σ^2^(*F*_o_^2^) + (0.0956*P*)^2^ + 0.2245*P*] where *P* = (*F*_o_^2^ + 2*F*_c_^2^)/3
3128 reflections	(Δ/σ)_max_ < 0.001
181 parameters	Δρ_max_ = 0.27 e Å^−^^3^
0 restraints	Δρ_min_ = −0.18 e Å^−^^3^

### Special details

**Table:**

Geometry. All s.u.'s (except the s.u. in the dihedral angle between two l.s. planes) are estimated using the full covariance matrix. The cell s.u.'s are taken into account individually in the estimation of s.u.'s in distances, angles and torsion angles; correlations between s.u.'s in cell parameters are only used when they are defined by crystal symmetry. An approximate (isotropic) treatment of cell s.u.'s is used for estimating s.u.'s involving l.s. planes.
Refinement. Refinement of *F*^2^ against ALL reflections. The weighted *R*-factor *wR* and goodness of fit *S* are based on *F*^2^, conventional *R*-factors *R* are based on *F*, with *F* set to zero for negative *F*^2^. The threshold expression of *F*^2^ > 2σ(*F*^2^) is used only for calculating *R*-factors(gt) *etc*. and is not relevant to the choice of reflections for refinement. *R*-factors based on *F*^2^ are statistically about twice as large as those based on *F*, and *R*- factors based on ALL data will be even larger.

### Fractional atomic coordinates and isotropic or equivalent isotropic displacement parameters (Å^2^)

**Table:**

	*x*	*y*	*z*	*U*_iso_*/*U*_eq_	
C1	0.6664 (3)	0.38694 (7)	0.77501 (14)	0.0320 (4)	
C2	0.7843 (3)	0.42644 (7)	0.69281 (14)	0.0309 (4)	
C3	0.6891 (3)	0.42666 (7)	0.57746 (15)	0.0328 (4)	
H3	0.7687	0.4530	0.5274	0.039*	
C4	0.3706 (3)	0.35103 (7)	0.60267 (14)	0.0307 (4)	
C5	0.4552 (3)	0.34821 (7)	0.72122 (14)	0.0310 (4)	
C6	0.3282 (4)	0.30618 (7)	0.78758 (16)	0.0381 (4)	
H6	0.3764	0.3023	0.8671	0.046*	
C7	0.1332 (4)	0.27070 (7)	0.73569 (16)	0.0398 (4)	
H7	0.0481	0.2426	0.7796	0.048*	
C8	0.0627 (3)	0.27700 (7)	0.61615 (15)	0.0349 (4)	
C9	1.0005 (3)	0.46984 (7)	0.72231 (14)	0.0330 (4)	
C10	1.2543 (4)	0.51876 (9)	0.87123 (18)	0.0525 (5)	
H10A	1.4364	0.5102	0.8395	0.079*	
H10B	1.2713	0.5202	0.9556	0.079*	
H10C	1.1896	0.5546	0.8418	0.079*	
C11	−0.1477 (4)	0.23821 (8)	0.55685 (18)	0.0448 (4)	
H11A	−0.2117	0.2543	0.4833	0.067*	
H11B	−0.3074	0.2329	0.6065	0.067*	
H11C	−0.0590	0.2025	0.5428	0.067*	
C12	0.3991 (4)	0.39784 (8)	0.40625 (15)	0.0421 (4)	
H12A	0.4415	0.4358	0.3801	0.051*	
H12B	0.1945	0.3926	0.3994	0.051*	
C13	0.5409 (5)	0.35647 (9)	0.32742 (18)	0.0560 (6)	
H19A	0.4711	0.3616	0.2479	0.084*	
H19B	0.4995	0.3188	0.3527	0.084*	
H19C	0.7430	0.3625	0.3311	0.084*	
N1	0.1777 (3)	0.31671 (6)	0.55030 (12)	0.0346 (3)	
N2	0.4905 (3)	0.39199 (6)	0.53125 (12)	0.0331 (3)	
O1	0.7306 (3)	0.38354 (6)	0.88110 (11)	0.0449 (4)	
O2	1.1179 (3)	0.49770 (6)	0.64973 (12)	0.0529 (4)	
O3	1.0520 (3)	0.47558 (6)	0.83641 (11)	0.0483 (4)	
O4	0.2506 (4)	0.38795 (8)	0.03949 (15)	0.0678 (5)	
H4B	0.099 (7)	0.3912 (13)	−0.025 (3)	0.102*	
H4A	0.394 (7)	0.3865 (14)	−0.003 (3)	0.102*	

### Atomic displacement parameters (Å^2^)

**Table:**

	*U*^11^	*U*^22^	*U*^33^	*U*^12^	*U*^13^	*U*^23^
C1	0.0303 (8)	0.0350 (9)	0.0306 (8)	0.0031 (6)	−0.0017 (6)	0.0001 (7)
C2	0.0285 (8)	0.0320 (8)	0.0322 (8)	0.0010 (6)	−0.0005 (6)	−0.0023 (7)
C3	0.0334 (8)	0.0311 (8)	0.0339 (9)	−0.0009 (6)	0.0000 (6)	0.0004 (7)
C4	0.0294 (8)	0.0304 (8)	0.0321 (8)	0.0033 (6)	−0.0007 (6)	−0.0006 (6)
C5	0.0301 (8)	0.0305 (8)	0.0324 (8)	0.0020 (6)	0.0002 (6)	0.0006 (7)
C6	0.0403 (9)	0.0399 (10)	0.0341 (9)	−0.0007 (7)	0.0005 (7)	0.0025 (7)
C7	0.0406 (9)	0.0368 (9)	0.0421 (10)	−0.0045 (7)	0.0036 (7)	0.0030 (8)
C8	0.0291 (8)	0.0325 (8)	0.0433 (9)	0.0011 (6)	0.0029 (7)	−0.0062 (7)
C9	0.0301 (8)	0.0349 (9)	0.0338 (9)	0.0015 (7)	−0.0019 (6)	−0.0006 (7)
C10	0.0581 (12)	0.0563 (12)	0.0427 (11)	−0.0220 (10)	−0.0066 (9)	−0.0083 (9)
C11	0.0428 (10)	0.0412 (10)	0.0505 (11)	−0.0069 (8)	0.0010 (8)	−0.0079 (8)
C12	0.0480 (10)	0.0434 (10)	0.0342 (9)	−0.0084 (8)	−0.0126 (8)	0.0078 (8)
C13	0.0709 (14)	0.0609 (13)	0.0361 (10)	−0.0118 (11)	−0.0036 (9)	−0.0068 (9)
N1	0.0317 (7)	0.0341 (7)	0.0377 (8)	−0.0003 (6)	−0.0017 (6)	−0.0028 (6)
N2	0.0348 (7)	0.0347 (8)	0.0296 (7)	−0.0011 (6)	−0.0041 (5)	0.0011 (6)
O1	0.0472 (7)	0.0558 (8)	0.0313 (7)	−0.0130 (6)	−0.0069 (5)	0.0055 (6)
O2	0.0583 (8)	0.0615 (9)	0.0388 (7)	−0.0266 (7)	−0.0020 (6)	0.0047 (6)
O3	0.0571 (8)	0.0544 (8)	0.0331 (7)	−0.0227 (6)	−0.0046 (6)	−0.0034 (6)
O4	0.0642 (10)	0.0912 (13)	0.0477 (9)	−0.0087 (9)	−0.0003 (8)	0.0004 (8)

### Geometric parameters (Å, º)

**Table:**

C1—O1	1.237 (2)	C9—O3	1.319 (2)
C1—C2	1.445 (2)	C10—O3	1.445 (2)
C1—C5	1.472 (2)	C10—H10A	0.9600
C2—C3	1.373 (2)	C10—H10B	0.9600
C2—C9	1.478 (2)	C10—H10C	0.9600
C3—N2	1.340 (2)	C11—H11A	0.9600
C3—H3	0.9300	C11—H11B	0.9600
C4—N1	1.344 (2)	C11—H11C	0.9600
C4—N2	1.395 (2)	C12—N2	1.479 (2)
C4—C5	1.395 (2)	C12—C13	1.498 (3)
C5—C6	1.395 (2)	C12—H12A	0.9700
C6—C7	1.366 (2)	C12—H12B	0.9700
C6—H6	0.9300	C13—H19A	0.9600
C7—C8	1.397 (2)	C13—H19B	0.9600
C7—H7	0.9300	C13—H19C	0.9600
C8—N1	1.328 (2)	O4—H4B	1.01 (4)
C8—C11	1.497 (2)	O4—H4A	0.84 (3)
C9—O2	1.203 (2)		
			
O1—C1—C2	125.82 (15)	H10A—C10—H10B	109.5
O1—C1—C5	120.43 (15)	O3—C10—H10C	109.5
C2—C1—C5	113.75 (14)	H10A—C10—H10C	109.5
C3—C2—C1	119.95 (14)	H10B—C10—H10C	109.5
C3—C2—C9	114.64 (14)	C8—C11—H11A	109.5
C1—C2—C9	125.38 (14)	C8—C11—H11B	109.5
N2—C3—C2	125.18 (15)	H11A—C11—H11B	109.5
N2—C3—H3	117.4	C8—C11—H11C	109.5
C2—C3—H3	117.4	H11A—C11—H11C	109.5
N1—C4—N2	116.27 (14)	H11B—C11—H11C	109.5
N1—C4—C5	124.62 (15)	N2—C12—C13	113.02 (16)
N2—C4—C5	119.10 (14)	N2—C12—H12A	109.0
C6—C5—C4	116.23 (15)	C13—C12—H12A	109.0
C6—C5—C1	121.03 (15)	N2—C12—H12B	109.0
C4—C5—C1	122.75 (14)	C13—C12—H12B	109.0
C7—C6—C5	119.97 (16)	H12A—C12—H12B	107.8
C7—C6—H6	120.0	C12—C13—H19A	109.5
C5—C6—H6	120.0	C12—C13—H19B	109.5
C6—C7—C8	119.43 (16)	H19A—C13—H19B	109.5
C6—C7—H7	120.3	C12—C13—H19C	109.5
C8—C7—H7	120.3	H19A—C13—H19C	109.5
N1—C8—C7	122.34 (15)	H19B—C13—H19C	109.5
N1—C8—C11	117.19 (16)	C8—N1—C4	117.42 (14)
C7—C8—C11	120.48 (16)	C3—N2—C4	119.21 (14)
O2—C9—O3	122.85 (15)	C3—N2—C12	119.92 (14)
O2—C9—C2	123.56 (15)	C4—N2—C12	120.87 (13)
O3—C9—C2	113.58 (14)	C9—O3—C10	116.29 (14)
O3—C10—H10A	109.5	H4B—O4—H4A	99 (3)
O3—C10—H10B	109.5		
			
O1—C1—C2—C3	−178.42 (16)	C3—C2—C9—O2	−10.8 (2)
C5—C1—C2—C3	2.3 (2)	C1—C2—C9—O2	171.14 (17)
O1—C1—C2—C9	−0.5 (3)	C3—C2—C9—O3	168.67 (15)
C5—C1—C2—C9	−179.75 (14)	C1—C2—C9—O3	−9.3 (2)
C1—C2—C3—N2	−0.6 (2)	C7—C8—N1—C4	0.8 (2)
C9—C2—C3—N2	−178.77 (15)	C11—C8—N1—C4	−178.85 (14)
N1—C4—C5—C6	0.4 (2)	N2—C4—N1—C8	179.33 (14)
N2—C4—C5—C6	−179.72 (14)	C5—C4—N1—C8	−0.8 (2)
N1—C4—C5—C1	−179.66 (14)	C2—C3—N2—C4	−1.5 (2)
N2—C4—C5—C1	0.2 (2)	C2—C3—N2—C12	177.86 (16)
O1—C1—C5—C6	−1.5 (2)	N1—C4—N2—C3	−178.45 (14)
C2—C1—C5—C6	177.77 (14)	C5—C4—N2—C3	1.7 (2)
O1—C1—C5—C4	178.56 (15)	N1—C4—N2—C12	2.2 (2)
C2—C1—C5—C4	−2.1 (2)	C5—C4—N2—C12	−177.68 (15)
C4—C5—C6—C7	0.0 (2)	C13—C12—N2—C3	99.01 (19)
C1—C5—C6—C7	−179.89 (16)	C13—C12—N2—C4	−81.6 (2)
C5—C6—C7—C8	0.0 (3)	O2—C9—O3—C10	1.6 (3)
C6—C7—C8—N1	−0.4 (3)	C2—C9—O3—C10	−177.90 (15)
C6—C7—C8—C11	179.24 (16)		

### Hydrogen-bond geometry (Å, º)

**Table:**

*D*—H···*A*	*D*—H	H···*A*	*D*···*A*	*D*—H···*A*
O4—H4*B*···O1^i^	1.01 (4)	2.02 (4)	2.994 (2)	163 (3)
O4—H4*A*···O1^ii^	0.84 (3)	2.09 (3)	2.928 (2)	176 (3)
O4—H4*B*···O3^i^	1.01 (4)	2.56 (3)	3.224 (2)	124 (2)
C3—H3···O2^iii^	0.93	2.40	3.293 (2)	160
C11—H11*C*···O4^iv^	0.96	2.59	3.539 (3)	168

Symmetry codes: (i) *x*−1, *y*, *z*−1; (ii) *x*, *y*, *z*−1; (iii) −*x*+2, −*y*+1, −*z*+1; (iv) *x*, −*y*+1/2, *z*+1/2.
